# Stimulation of the Nigrotectal Pathway at the Level of the Superior Colliculus Reduces Threat Recognition and Causes a Shift From Avoidance to Approach Behavior

**DOI:** 10.3389/fncir.2018.00036

**Published:** 2018-05-07

**Authors:** Rafael C. Almada, Andreas J. Genewsky, Daniel E. Heinz, Paul M. Kaplick, Norberto C. Coimbra, Carsten T. Wotjak

**Affiliations:** ^1^Department of Stress Neurobiology and Neurogenetics, Neuronal Plasticity, Max Planck Institute of Psychiatry, Munich, Germany; ^2^Laboratory of Neuroanatomy and Neuropsychobiology, Department of Pharmacology, Ribeirão Preto Medical School (FMRP), University of São Paulo (USP), São Paulo, Brazil; ^3^Behavioral Neurosciences Institute (INeC), São Paulo, Brazil; ^4^Neuroscience Master’s Program, Interdisciplinary Center for Neurosciences (IZN), Heidelberg University, Heidelberg, Germany; ^5^NAP-USP-Neurobiology of Emotions Research Center (NuPNE), Ribeirão Preto Medical School (FMRP), University of São Paulo (USP), São Paulo, Brazil

**Keywords:** substantia nigra pars reticulata (SNr), deep layers of superior colliculus (dlSC), nigrotectal pathway, robo-beetle, fear, optogenetics, beetle mania task (BMT)

## Abstract

Defensive behavioral responses are essential for survival in threating situations. The superior colliculus (SC) has been implicated in the generation of defensive behaviors elicited by visual, tactile and auditory stimuli. Furthermore, substantia nigra pars reticulata (SNr) neurons are known to exert a modulatory effect on midbrain tectum neural substrates. However, the functional role of this nigrotectal pathway in threating situations is still poorly understood. Using optogenetics in freely behaving mice, we activated SNr projections at the level of the SC, and assessed consequences on behavioral performance in an open field test (OFT) and the beetle mania task (BMT). The latter confronts a mouse with an erratic moving robo-beetle and allows to measure active and passive defensive responses upon frequent encounter of the threatening object. Channelrhodopsin-2 (ChR2)-mediated activation of the inhibitory nigrotectal pathway did not affect anxiety-like and exploratory behavior in the OFT, but increased the number of contacts between robo-beetle and test mouse in the BMT. Depending on the size of the arena, active avoidance responses were reduced, whereas tolerance and close following of the robo-beetle were significantly increased. We conclude from the data that the nigrotectal pathway plays holds the potential to modulate innate fear by attenuating threat recognition and causing a shift from defensive to approach behavior.

## Introduction

The ability to sense and predict threatening or stressful events is essential for survival. Accordingly, the brain has developed distinct pathways to control and process different types of fear (Gross and Canteras, 2012; Tovote et al., 2015). While the hippocampus, the amygdala, and the prefrontal cortex play a fundamental role in conditioned fear (Sotres-Bayon et al., 2012; Tovote et al., 2015), mesencephalic structures, such as the superior colliculus (SC) and the periaqueductal gray (PAG), are part of a complex neuronal circuit underlying innate defensive responses (Coimbra and Brandão, 1993; Brandão et al., 1999). For instance, electrical activation of the SC was found to cause an increase in defensive behavior, such as alertness, freezing and escape, along with autonomic responses (Brandão et al., 1994). More recently, it could be demonstrated that optogenetic activation of parvalbumin-positive SC neurons triggers both active (avoidance) and passive (freezing) fear responses depending on stimulus properties and sex of the mice (Shang et al., 2015). The SC receives multiple sensory inputs—of visual (Hikosaka and Wurtz, 1983; Feinberg and Meister, 2014; Shi et al., 2017), auditory (King, 2004) and tactile (Favaro et al., 2011) nature—which predisposes it as a central hub for translating sensory information into innate defensive responses (Wei et al., 2015).

SC activity is tightly controlled by GABAergic signaling (Brandão et al., 1994). For instance, local infusion of the GABA_A_ receptor antagonist bicuculline was observed to cause patterns of defensive responses, as from electrical stimulation (Brandão et al., 2005). Even though we cannot entirely rule out the involvement of local GABAergic interneurons, there is evidence for a significant contribution of GABAergic afferences from the substantia nigra pars reticulata (SNr) during the expression of innate fear-related responses (Castellan-Baldan et al., 2006). First, anterograde tracing revealed a dense projection from the SNr to deep layers of the SC (dlSC; Ribeiro et al., 2005), the so-called nigrotectal pathway, which additionally innervates the dorsal PAG (Jayaraman et al., 1977; Grofová et al., 1978). Second, this nigrotectal pathway is primarily comprised by GABAergic neurons (Ribeiro et al., 2005), and GABAergic cells in the SNr tonically inhibit neural firing of dlSC (Hikosaka and Wurtz, 1983; Grillner and Robertson, 2016; Hormigo et al., 2016). Third, inactivation of neuronal somata of the SNr increased escape behavior which was elicited by microinjections of the GABA_A_ receptor antagonist bicuculline in the dlSC (Almada and Coimbra, 2015). Moreover, chemogenetic and optogenetic manipulations of GABAergic neurons at the level of the SNr have promoted (in case of inhibited neuronal activity) respectively attenuated (in case of enhanced neuronal activity) active avoidance in an auditory-cued conditioning paradigm (Hormigo et al., 2016).

Despite compelling evidence for anatomical, physiological and functional interactions between the SNr and dlSC, direct demonstration of an involvement of nigrotectal projections in modulation of innate defensive responses is still missing. This might be ascribed to the lack of appropriate animal models, which allow to study the whole bandwidth of fear reactions. Therefore, we employed a recently established behavioral paradigm that enables the quantification of both, passive and active defensive responses, upon frequent encounters with an approaching robo-beetle (Heinz et al., 2017). Using this task, we investigated the consequences of optogenetically activating afferences from the SNr, directly at the level of the dlSC during the confrontation with the approaching robo-beetle.

## Materials and Methods

### Animals

Experiments were performed with male C57BL/6N mice purchased from Charles River (Bad Sulzfeld, Germany) aged 8–15 weeks. All mice were naïve before surgery and maintained on a 12 h:12 h inverted light cycle (lights off: 08:00 h) under standard housing conditions (23 ± 4°C, 50 ± 10% humidity) in type 2 macrolon cages (groups of four per cage) with *ad libitum* access to food (1314, Altromin Spezialfutter GmbH & Co. KG, Lage, Germany) and water. All behavioral tests were carried out in the dark phase between 09:00 h and 17:00 h. Experimental procedures were performed according to the European Community Council Directive 2010/63/EEC and approved by the local government of Upper Bavaria (55.2.1.54-2532-142-12, 55.2.1.54-2532-188-12 and 55.2.1.54-2532-08-16). Efforts were made to minimize animal suffering and to reduce the number of animals used in the present work.

### Viral Injections and Optogenetics

Mice were treated with an analgesic (Vetalgin, 200 mg/kg), anasthetized with isoflurane (Forene^®^, Abbott, Germany, under induction at 4%, maintained at 1.5%), and headfixed in a stereotaxic frame (Leica Biosystems, Nussloch, Germany, AngleTwo). Craniotomies were made bilaterally and the stereotaxic coordinates used for SNr were AP −3.2 mm, ML ±1.5 mm, DV −4.2 mm, from the skull surface. For SNr→SC stimulation, 350 nl of AAV5-hSyn-Channelrhodopsin-2 (ChR2; H134R)-mCherry (ChR2; *n* = 10) or the control vector AAV5-hSyn-mCherry (mCherry; *n* = 8) was bilaterally injected using a Hamilton syringe (80 nl/min) into the SNr and mice were randomly assigned to ChR2 or mCherry groups. All viral aliquots were obtained from the University of North Carolina Vector Core (Chapel Hill, NC, USA). For optical manipulation, fiber optic cannulas (Thorlabs, Dachau/Munich, Germany, CFML12L10, Ø200 μm, NA 0.39, cut to 3 mm length) were implanted targeting the dlSC (AP: −3.9 mm, ML: ± 1.1 mm, DV: −2.0 mm) 5–6 weeks after the virus injections. The fiber tip was lowered at an angle of 12°C. The mice were allowed to recover for 2 weeks after the fiber implantation. For optical stimulation of ChR2, pulsed (5 ms at 20 Hz) laser light (Omicron-Laserage, Rodgau-Dudenhofen, Germany, LightHUB-4) of 460 nm was applied. The laser output power was set to measure 7.5 mW at the single fiber tip. Bilateral optical stimulation was achieved using a fiber-optic rotary joint with two output ports (Doric Lenses Inc., Quebec, QC, Canada, FRJ_1x2i_FC-2FC).

### Behavioral Tests

#### Open Field Test (OFT)

The open field arena consisted of a gray Plexiglas cube (L50 × W50 × H53 cm), which was divided into a center zone (25 × 25 cm) and an outer peripheral zone (Figure 4B). Mice were connected to the fiber-optic cables, placed in the center, and allowed 3 min to recover from handling before assessment for 9 min. The OFT session was divided into three 3-min epochs with alternating laser manipulation (OFF–ON–OFF; Felix-Ortiz et al., 2016). Video tracking (Any-maze, Stoelting, Dublin, Ireland) was employed to track the location and locomotion of the mouse in the open field. All measurements were quantified relative to the mouse center. The setup was illuminated with 50 Lux white light.

**Figure 1:**
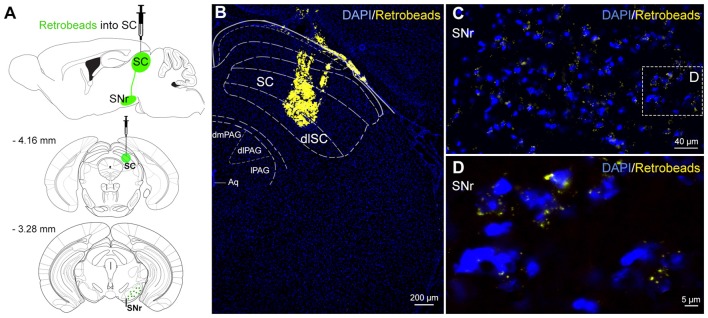
Neural connections from substantia nigra pars reticulata (SNr) to superior colliculus (SC). **(A)** Schematic representation of anatomical tracing strategy. Retrobeads were unilaterally injected into the SC (*n* = 4). **(B)** The fluorescent latex beads injected into the SC were retrogradely transported to SNr neurons **(C,D)**.

**Figure 2:**
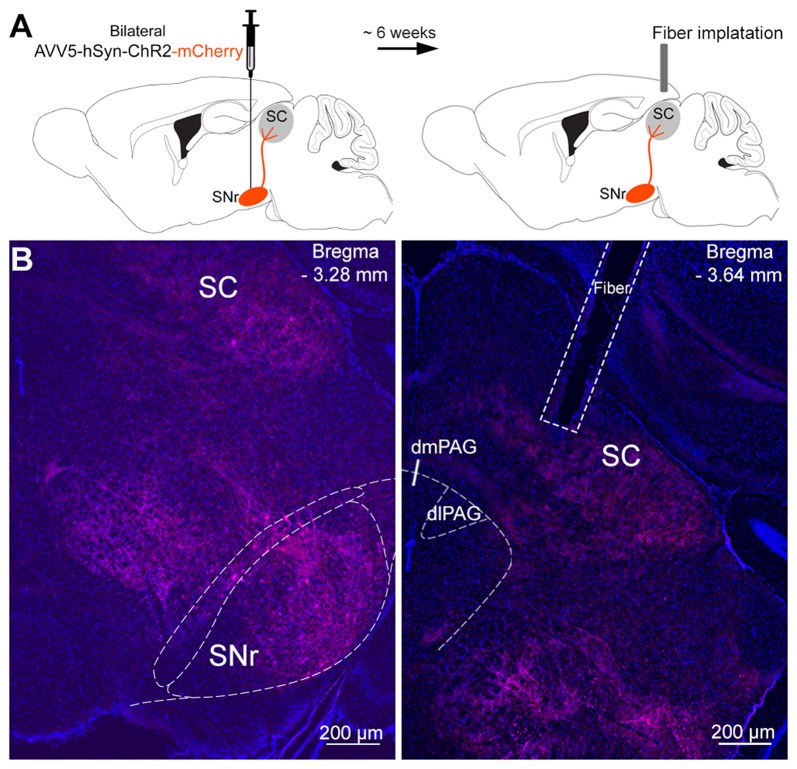
Infusion of viral vectors into the SNr. **(A)** Viral vectors were injected into the SNr, resulting in the expression of either Channelrhodopsin-2 (ChR2)-mCherry (*n* = 10) or mCherry (*n* = 8) in the nigrotectal pathway. The optical fibers were placed above the SC.** (B)** Representative coronal photomicrographs showing the expression of ChR2-tdtomato in SNr somata, as well as in SNr terminals within the periaqueductal gray (PAG) matter and SC (blue: DAPI, red: tdtomato).

**Figure 3:**
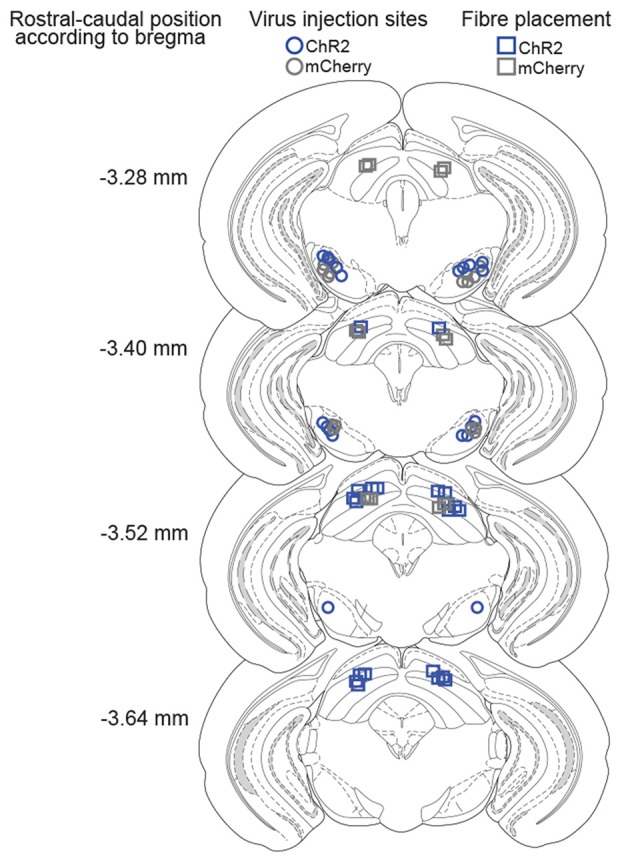
Histological verification of injection and implantation sites. Coronal drawings across rostro-caudal extensions of the SNr and SC, depicting the center of viral infusions in the SNr (ChR2-mice: blue circles, mCherry-mice: gray circles) and fiber placements in the SC (ChR2-mice: blue squares, mCherry-mice: gray squares).

**Figure 4:**
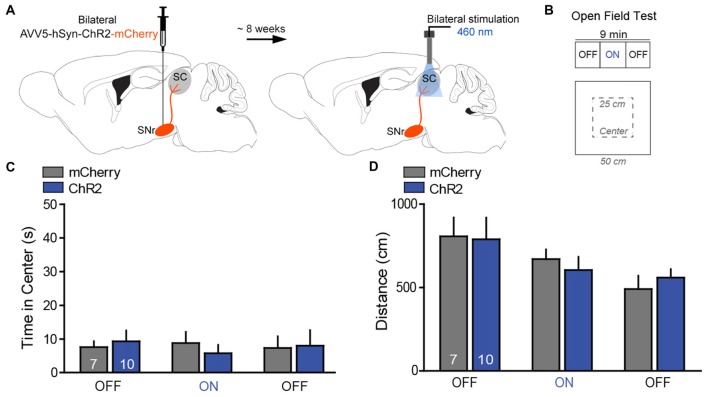
Photostimulation of SNr→SC projections at the level of the SC did not alter anxiety-like behavior or locomotor activity.** (A)** Design of the optogenetic approach with transfection of the SNr either with ChR2-mCherry (*n* = 10) or mCherry (*n* = 8) and placement of the optical fibers above projection terminals of the nigrotectal pathway at the level of the SC. **(B)** The open field test (OFT) consisted of 3-min epochs with alternating laser treatment (OFF–ON–OFF).** (C)** Average time spent in exploring the center of the OFT arena. ChR2-mice did not show significant differences regarding the time spent in the center of the arena during the ON epoch, relative to mCherry-mice and the OFF epochs.** (D)** No significant effects were detected for the total distance traveled in the OFT. Data are shown as mean ± standard error of the mean (SEM). Numbers within bars indicate the number of mice per group.

#### Beetle Mania Task (BMT)

The beetle mania task (BMT) has been developed and validated to enable the assessment of both passive and, in particular, active fear responses (Heinz et al., 2017). Experiment 1 was performed in a large arena (L150 × W15 × H37 cm) made out of gray polyethylene as previously described (Heinz et al., 2017). In Experiment 2 which was performed 24 h later, the size of the arena was reduced to 1/3 (L50 × W15 × H37 cm). In both experiments the arena was illuminated with 50 Lux white light and was divided into equally spaced segments of 25 cm. The BMT is comprised of two successive 5-min phases: during the habituation phase, mice were acclimatized to the testing arena. Vertical exploration (number of rearings) and locomotion (number of segment crossings) were scored online by an experienced observer, blind to the experimental condition. The habituation phase was subsequently followed by the test phase with unpredictable confrontations with the erratically moving robo-beetle (Hexbug Nano^1^, Innovation First Labs Inc., Greenville, TX, USA; L4.5 × W1.5 × H1.8 cm, weight: 7.3 g, mean speed: 25 cm/s). In the testing phase, we placed the robot-beetle far most distant from the mouse, and scored the following behavioral measures on-line: *chasing contacts* (number of physical contacts between robo-beetle and mouse), *approach* (number of sectors the mouse follows the by-passing robo-beetle in close vicinity), *tolerance* (ignorance of the approaching robo-beetle, normalized to chasing contacts), *avoidance* behavior as the sum of *escape response* (the mouse withdrawals with accelerated speed in direction of the beetle’s movement vector, this reaction does not require physical contact between robo-beetle and mouse) and *flight response* (the mouse withdrawals with accelerated speed in the direction opposite to the beetle’s movement vector; Heinz et al., 2017) normalized to total contacts, and *freezing* (freezing behavior during the complete test phase; offline analysis).

Laser stimulation was performed only in test phases. In Experiment 1, the laser was activated when the robo-beetle was in the same segment or the adjacent segment as the mouse. The offset of the stimulation was triggered if either the robo-beetle or the mouse has left the “critical segments.” The number of *laser events* was recorded. In Experiment 2, the laser was activated during the whole test phase.

### Anatomical Tracing

To identify the origin of SNr inputs to SC, we injected 350 nl of the retrogradely transported fluorescent latex beads (retrobeads, Lumafluor) into the SC (AP: −3.5 mm; ML: ±1.0; DV: −2.2 mm) in C57BL/6N mice. Four days after injection, mice were sacrificed, transcardially perfused with 4% paraformaldehyde in PBS, and brains were extracted and processed for histology as described below. Brains were sliced into 40 μm coronal sections by cryostat (Leica CM 3000, Wetzlar, Germany) and co-stained with DAPI.

### Histology

Following completion of experiments, mice were sacrificed with an overdose of isoflurane immediately transcardially perfused with ice cold PBS followed by 4% paraformaldehyde. Brains were post-fixed overnight at 4°C in paraformaldehyde and then cryoprotected with 30% sucrose in PBS. For optogenetic experiments, brains were sliced at 40 μm with a cryostat. The locations of electrodes, fibers and injection sites were compared with the atlas of Franklin and Paxinos (2008). The experimenter was always blind to the behavioral results in the histological analysis.

### Statistical Analysis

Data are presented as mean ± standard error of the mean (SEM) or as individual data with means ± SEM. Statistical analyses were performed as indicated in the results section using Graphpad Prism (version 6.0; GraphPad Software Inc., San Diego, CA, USA). Significance was accepted if *P* ≤ 0.05; significance levels are indicated as follows: (*)*P* < 0.05; (**)*P* < 0.01.

## Results

### Nigrotectal Projections: Retrograde Tracing

To visualize the origin of the nigrotectal pathway for subsequent optogenetic experiments, we injected retrogradely transported green fluorescent latex beads into the dlSC (Figure 1A). Retrobeads which were taken up from projection terminals within the dlSC (Figure 1B), were retrogradely transported and found throughout the entire SNr (Figures 1C,D). This indicates a homogeneous distribution of dlSC-projecting neurons within the SNr. Therefore, there was no reason to restrict injections of viral vectors (see next paragraph) to distinct subregions of the SNr.

### Stimulation of SNr Projections at the Level of the SC Did Not Exert Anxiolytic Effects

The SNr was bilaterally transfected with viral vectors encoding for ChR2-mCherry or mCherry only (controls), under control of the hSyn promoter (Figure 2A). Six weeks later, fibers stubs were implanted directly above the projection terminals of the nigrotectal pathway within the dlSC (Figure 2A), and the animals were allowed to recover for 2 weeks. Placements of injection and implantation sites (for representative photographs see Figure 2B) were verified in the end of the study, and only mice with adequate placement of the injection sites and optical fibers were incluided into analyses (Figure 3).

In the OFT, photostimulation of the nigrotectal pathway (Figures 4A,B) failed to affect center time (Figure 4C; 2-way ANOVA for repeated measures; group: *F*_(1,15)_ = 0.02, *P* = 0.95; laser: *F*_(1,15)_ = 0.09, *P* = 0.91; interaction: *F*_(1,15)_ = 0.39, *P* = 0.68) and distance traveled (Figure 4D; group: *F*_(1,15)_ = 0.02, *P* = 0.96; laser: *F*_(1,15)_ = 0.65, *P* = 0.59; interaction: *F*_(1,15)_ = 0.38, *P* = 0.67). These findings indicate that activation of SNr→SC projections at the level of the SC did not alter anxiety-like behavior and locomotor activity.

### Activation of SNr Afferences at the Level of the SC Decreases Threat Recognition and Promotes Tolerance in the Beetle Mania Task (BMT)

**Figure 5:**
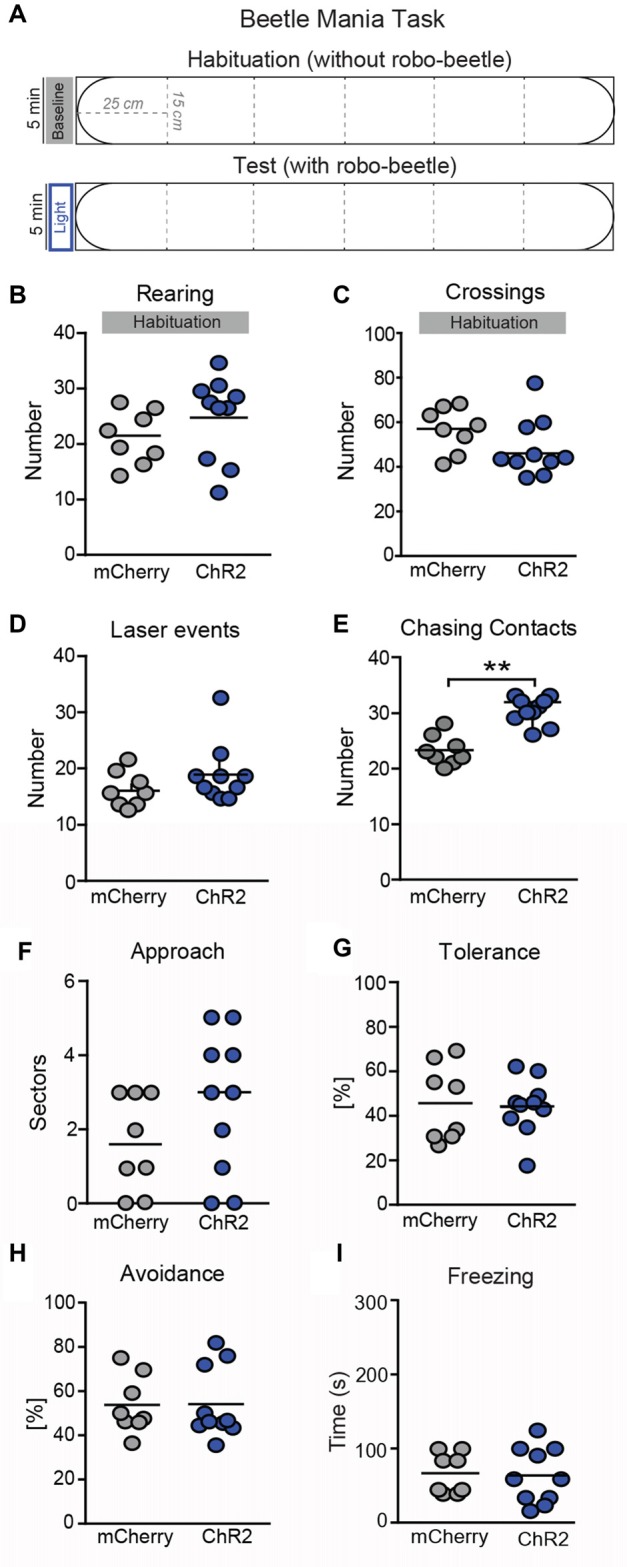
Photostimulation of SNr→SC projections at the level of the SC decreases threat recognition in the beetle mania task (BMT). **(A)** The BMT consisted of two consecutive 5-min phases in an arena (L150 × W15 × H37 cm). In the habituation phase, mice were allowed to habituate to the arena without robo-beetle or optical stimulations. The test phase included confrontations with an erratic moving robo-beetle and laser stimulations. The laser was active if the beetle had entered the sector adjacent to the mouse or the mouse sector. No significant effects were detected in **(B)** vertical exploration (rearings) or **(C)** locomotor activity (segment crossings) during habituation phase. In the following test phase, no significant differences were observed in **(D)** number of laser events, but **(E)** number of chasing contacts. No significant effects were detected in **(F)** approach behavior, **(G)** tolerance (expressed as a percentage of contacts), **(H)** avoidance (expressed as a percentage of contacts) and **(I)** freezing. **P < 0.01.

To study consequences of increased activity in SNr→SC projections on defensive behavior, we tested animals in the BMT. In this test, mice are confronted with an erratically moving, potentially threatening robo-beetle (Heinz et al., 2017). During habituation to the setup when no laser stimulation was employed (Figures 5B,C), both groups of mice showed the same exploratory behavioral activity (Figure 5B; *t*_(16)_ = 0.173, *P* = 0.86; Figure 5C; *t*_(16)_ = 0.354, *P* = 0.77) and no significant differences were observed in case of laser events (Figure 5D; *t*_(16)_ = 1.24, *P* = 0.23). In the subsequent test phase, mice were confronted with the beetle, and laser stimulation was activated when the beetle was in the same segment as the mouse or the adjacent segments (Figure 5A). Stimulation of SNr→SC projections at the level of the SC selectively increased the number of chasing contacts between the beetle and the mouse (Figure 5E; *t*_(16)_ = 5.77, *P* = 0.003), whereas no significant differences were observed on approach (Figure 5F; *t*_(16)_ = 0.92, *P* = 0.37), tolerance (Figure 5G; *t*_(16)_ = 0.37, *P* = 0.71), avoidance (Figure 5H; *t*_(16)_ = 0.058, *P* = 0.955), and freezing behavior (Figure 5I; *t*_(16)_ = 1.55, *P* = 0.86).

**Figure 6:**
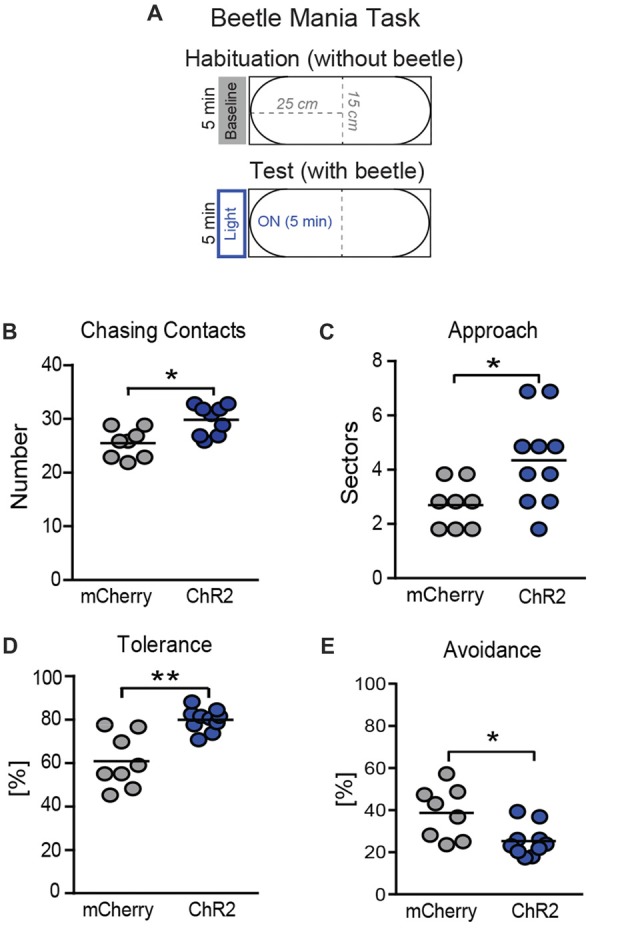
Consequences of photostimulation of SNr→SC projections at the level of the SC on threat recognition in a smaller arena.** (A)** The BMT was repeated in a smaller arena (length: 50 cm), again with 5 min of habituation without beetle or light stimulation, followed by the 5-min test phase with the robo-beetle and laser stimulation being present throughout the entire phase. Stimulation of axon terminals of SNr neurons at the level of the SC increased **(B)** the number of chasing contacts, **(C)** approach behavior and **(D)** tolerance behavior (expressed as a percentage of contacts), while decreasing **(E)** avoidance behavior bars indicate the number of mice per group. *P < 0.05, **P < 0.01.

Further, we tested how activation of the SNr→SC pathway affects the interaction between the mouse and the beetle in a test situation with higher emotional load. To this end, we confronted the animals with the robo-beetle in a smaller arena (Figure 6A) with laser stimulation throughout the entire 5-min test phase (Figure 6A). Again, stimulation of the SNr-SC pathway at level of the SC increased the number of chasing contacts (Figure 6B; *t*_(16)_ = 2.92, *P* = 0.012). This time, however, we additionally observed an increase in approach behavior (Figure 6C; *t*_(16)_ = 2.09, *P* = 0.05) and tolerance (Figure 6D; *t*_(16)_ = 3.28, *P* = 0.006) and a decrease in avoidance behavior (Figure 6E; *t*_(16)_ = 2.88, *P* = 0.011). We could confirm the apparent shift in the behavioral responses to the robo-beetle from defensive to approach behavior by calculating the ratio of approach and avoidance behavior for each mouse (mCherry: 0.34 ± 0.05; ChR2: 0.65 ± 0.06; *t*_(16)_ = 3.93, *P* = 0.001).

## Discussion

Using optogenetics in combination with the recently established ethobehavioral BMT, we provide first direct evidence that activation of the nigrotectal pathway at the level of the SC reduces threat recognition and active avoidance, while increasing tolerance and approach behavior towards an erratic moving robo-beetle, without affecting exploratory behavior in an open field.

Our results confirm and extend previous reports about the importance of the nigrotectal pathway for the control of innate defensive behaviors (Coimbra and Brandão, 1993; Ribeiro et al., 2005; Almada and Coimbra, 2015; Almada et al., 2015; Hormigo et al., 2016). The SNr sends projections not exclusively to the SC, but to a variety of brain structures (Grillner and Robertson, 2016). Therefore, manipulations of the SNr (Hormigo et al., 2016; even with simultaneous manipulations of the SC; for reference see Almada and Coimbra, 2015; Almada et al., 2015; Hormigo et al., 2016) cannot unequivocally validate an involvement of SNr→SC projections in fear regulation. This can only be achieved by direct manipulations of projection terminals at the level of the SC. We used viral vectors to drive the expression of the light-activated cation channel ChR2 in SNr neurons, while stimulating axon terminals in the SC. One limitation of this approach is that we cannot exclude an antidromic activation of neurons at level of SNr, which might result in activity changes in collateral nigrothalamic and/or nigropontine projections as well (Mailly et al., 2003).

Behavioral consequences of the stimulation were surprisingly distinct, given previous reports about an involvement of the SC in several defensive behavioral responses, such active avoidance, panic-like explosive flight responses, escape and freezing (Almada and Coimbra, 2015; Almada et al., 2015). We observed an increase in the number of contacts between the approaching robo-beetle and the test animal and, depending on stimulation duration and the size of the test arena, also an increase in tolerance of the encounter (i.e., mice allowed the robo-beetle to bypass without showing freezing or avoidance responses) and a decrease in avoidance responses. These behavioral alterations could not be explained by a general reduction in locomotor activity, since laser stimulation failed to affect exploration of an open field and even caused an increase in approach behavior, whereby the mouse followed the bypassing robo-beetle. The increase in contacts is particularly remarkable, given the fact that the number of encounters with the robo-beetle is highly conserved among a variety of inbred and outbred mouse strains and resistant to pharmacological treatments with benzodiazepines or enhancers of endocannabinoid signaling (Heinz et al., 2017).

Stimulation of the SNr→SC projections failed to affect tolerance, avoidance and approach behavior in the large arena, while reducing defensive and enhancing offensive responses in the smaller arena. Therefore, activation of the SNr→SC projections holds the potential to affect both threat detection and responding by shifting the balance of avoidance vs. approach behavior depending on the experimental settings. It remains to be shown in future studies, whether the different consequences of ChR2 stimulation in the large vs. the small arena result from differences in: (i) emotional load of the test situation; (ii) familiarity with the test procedure; and/or (iii) duration of the laser stimulation. In any case, the absence of effects on avoidance responses in the large arena was unexpected, given the prominent role of the SC in initiating such defensive responses (Shang et al., 2015). In this context we have to consider that stimulation of neurons in the lateral SC evokes approach-like and appetitive movements, while stimulation of neurons medially situated in rostral midbrain tectum together with deep layers of the SC induces fear-related responses (Dean et al., 1989; Comoli et al., 2012). In case of the present study, however, we cannot ascribe the lack of effects in the large arena to interindividual differences in the placement of the optic fiber, since the same animals were tested in the large arena (wihtout effects) and the small arena (with effects on defensive and offensive behavior). Moreover, the SNr→SC projections seem to play a modulatory rather than instructive role for threat responses triggered at level of the SC. It is conceivable that the confronation with the robo-beetle in the large arena was insufficient in triggering panic-like behavior in regular C57BL/6N mice, which might be partially due to a sufficiently high* a priori* activity in the SNr→SC projections. Consequently, additional stimulation of the SNr→SC projections by optogenetic means would only affect defensive responses in more threatening situation, such as in the smaller arena. It might well be that similar interventions modulate fear-related behavior also in the large arena, if applied to mice with* a priori* higher levels of panic-like responses (see Heinz et al., 2017).

Compared to other ethobehavioral tasks, which are based on only a few confrontations with large robogators (Choi and Kim, 2010; Amir et al., 2015), the BMT allows the analysis of multiple encounters with an ambiguous, only potentially-threatening robo-beetle. Moreover, the dimension of the robo-beetle enables the assessment of both defensive and offensive responses. This is of particular interest for studies of the nigrotectal pathway, given the involvement of the SC in predatory hunting (Furigo et al., 2010; Comoli et al., 2012). Indeed, we could observe an increase in close following of the bypassing robo-beetle upon stimulation of the nigrotectal pathway, which inhibits neuronal activity at the level of the SC (Hormigo et al., 2016). At the first glance, this seems to contradict pervious observations according to which inactivation of the SC reduces predatory hunting (Furigo et al., 2010). Apparently, close-following as shown in social interactions is different from hunting behavior (Hoy et al., 2016; Han et al., 2017; Park et al., 2018). Taking into consideration that we failed to observe any catching, biting or pulling of the robo-beetle, we conclude that the increase in approach behavior observed in the present study most likely results from a devaluation of the threat associated with the bypassing beetle rather than an initiation of hunting.

The SC is a multimodal sensory-motor structure that receives inputs from the retina and somatosensory cortex (King, 2004; Shi et al., 2017). Thus, arguably, stimulation of the nigrotectal pathway, which results in inhibition of SC neurons, may obscure sensory perceptions. In this context we have to consider that the SC is separated into superficial, intermediate and deep layers (Redgrave et al., 1993; Shi et al., 2017). Although the superficial layers are involved in diverse visual response properties (Shi et al., 2017), including detection of moving stimuli changes (Savage et al., 2017), the deep layers are highly associated with defensive behaviors (Brandão et al., 1994). In this scenario, intrinsic connections from the superficial layers to the dlSC appear to provide a rapid route for orienting movements of the head and eyes (Redgrave et al., 1993; Shang et al., 2015) towards a given stimulus. It is known that the position of the head in space is essential for a variety of tasks, including defensive behaviors (Dean et al., 1989), among others (Wang and Redgrave, 1997; Furigo et al., 2010). This suggests that the SC acts as a crucial structure which is strongly implicated in initial behavioral responses to visual sensory events (Comoli et al., 2012), such as those related to threatening stimuli. Histological verification of the transfections could localize projection terminals of SNr neurons in the dlSC, where the optical fibers were aimed at. Therefore, stimulation of the nigrotectal pathway seemed to affect the translation of primary sensory perception into first-line defensive responses. Given the multisensory nature of the robo-beetle (i.e., its movement produces noise and vibrations in addition to its visual appearance), it is highly likely that the observed behavioral phenotype results from interference not only with visual, but also tactile and auditory signals emitted by the approaching robo-beetle. In this context it is of interest that a recent study could demonstrate not only an inhibitory tone of SNr→SC projections on whisker stimulation-evoked neuronal activity in the SC, but also a highly integrative role of the SNr in orchestrating auditory-cued active avoidance learning (Hormigo et al., 2016).

Taken together, we demonstrate that the nigrotectal pathway has the potential to control defensive responses to threatening stimuli. Specifically, projections from the SNr to deep layers of the SC appear to selectively dampen threat recognition and responding.

## Compliance with Ethical Standards

Animal experiments have been performed strictly following the 3R-rule (i.e., every effort was taken to keep the number of experimental subjects at a minimum and to ensure proper anesthesia and analgesia during and, in case of analgesia, after surgery). Experimental procedures have been approved by the local authorities (see “Materials and Methods” section). All authors have read and agreed about the manuscript.

## Author Contributions

RCA and AJG contributed equally to this work. RCA, AJG and CTW conceived the hypothesis, designed the experiments. RCA, AJG and DEH performed experiments. RCA, AJG, DEH and PMK acquired the data. RCA, AJG, DEH, NCC and CTW analyzed data and interpreted the results. RCA and CTW wrote the manuscript draft. All authors reviewed and approved the manuscript.

## Conflict of Interest Statement

The authors declare that the research was conducted in the absence of any commercial or financial relationships that could be construed as a potential conflict of interest.

## Footnotes

^1^www.hexbug.com/nano
